# Supplementary material for: Modular nonlinear hybrid plasmonic circuit
Source: Nat Commun. 2020 May 15;11:2413. doi: 10.1038/s41467-020-16190-z (PMC7229200; doi:10.1038/s41467-020-16190-z)
Supplement: Supplementary file 1 — Supplementary Information [file 41467_2020_16190_MOESM1_ESM.pdf]

Supplementry Information:
"Modular Nonlinear Hybrid Plasmonic Circuit"

Alessandro Tuniz,[1,2,*] Oliver Bickerton,[1] Fernando J. Diaz,[1] Thomas Käsebier,[3] Ernst-Bernhard Kley,[3] Stefanie Kroker,[4,5] Stefano Palomba,[1,2] and C. Martijn de Sterke[1,2]

[1]*Institute of Photonics and Optical Science, School of Physics, The University of Sydney, NSW 2006 Australia*

[2]*The University of Sydney Nano Institute, The University of Sydney, NSW 2006, Australia*

[3]*Institute of Applied Physics, Abbe Center of Photonics, Friedrich Schiller Universität Jena, Max-Wien-Platz 1, 07743 Jena, Germany*

[4]*Physikalisch-Technische Bundesanstalt, Bundesallee 100, 38116 Braunschweig, Germany*

[5]*Technische Universität Braunschweig, LENA Laboratory for Emerging Nanometrology, Pockelsstrasse 14, 38106 Braunschweig, Germany*

(Dated: March 27, 2020)

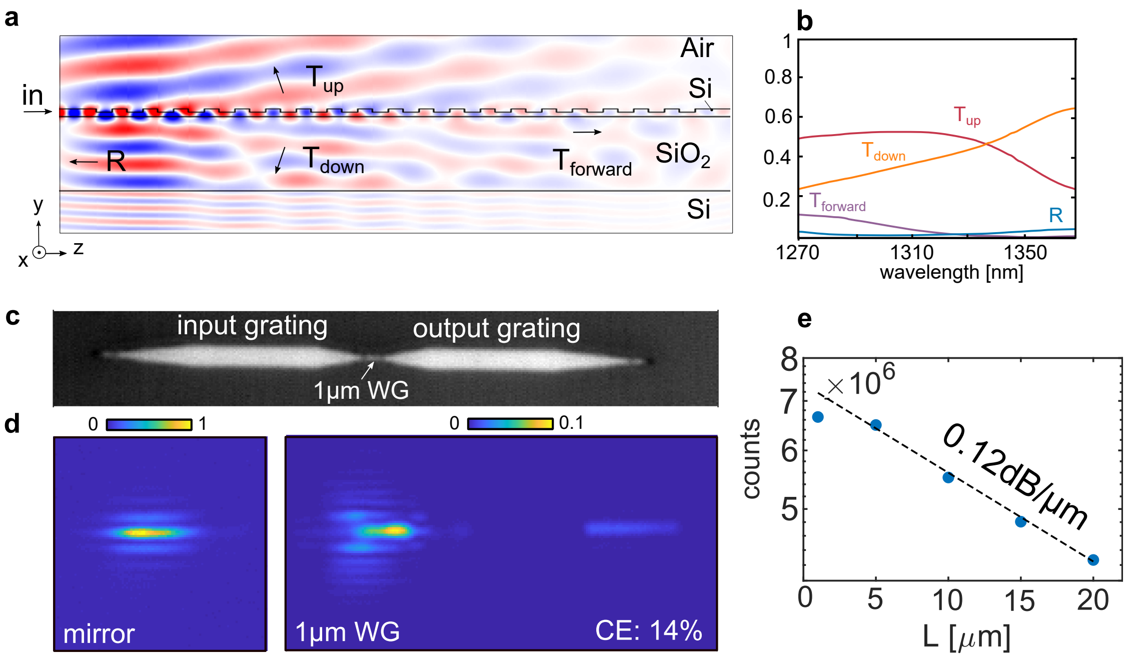

Supplementary Fig. 1. Silicon-on-insulator waveguide (SOI-WG) numerical design and experimentally measured properties. (a) Electric field for the SOI-WG calculations used to obtain the designed grating, with parameters as described in Methods. (b) Calculated parameters as a function of wavelength, as defined in (a). (c) Microscope image of a 1 μm waveguide and the input and output gratings. (d) Left: intensity profile of the incident elliptical beam incident on the input grating. Right: scattered light by the waveguide with optimized coupling. Colorbars represent the number of photon counts measured by the InGaAs camera, divided by the maximum. (e) Cutback measurements using different waveguides, resulting in a measured waveguide propagation loss of $0.12\,\mathrm{dB}/\mu\mathrm{m}$.

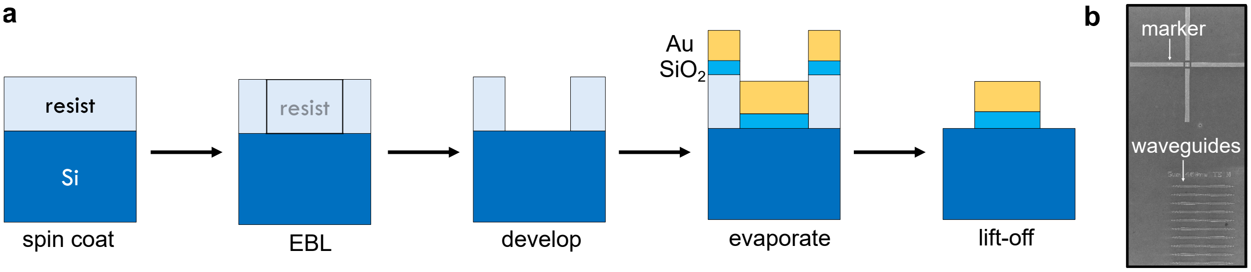

Supplementary Fig. 2. Fabrication process summary. (a) The waveguides are spin-coated with PMMA resist. The hybrid plasmonic integrated circuity (HPIC) structures are written with standard electron-beam lithography (EBL) and developed with Methyl isobutyl ketone. 20 nm silica and 50 nm gold are subsequently coated with electron-beam evaporation. Finally, a lift-off step (Methyl-isobutyl-ketone) removes the resist. The alignment precision ($\sim 10\,\text{nm}$) is obtained using local gold markers, placed in the immediate vicinity of our off-the-shelf waveguides. (b) Example scanning electron micrograph detail of waveguides and local gold marker (window size: $110 \times 240\,\mu\text{m}^2$).

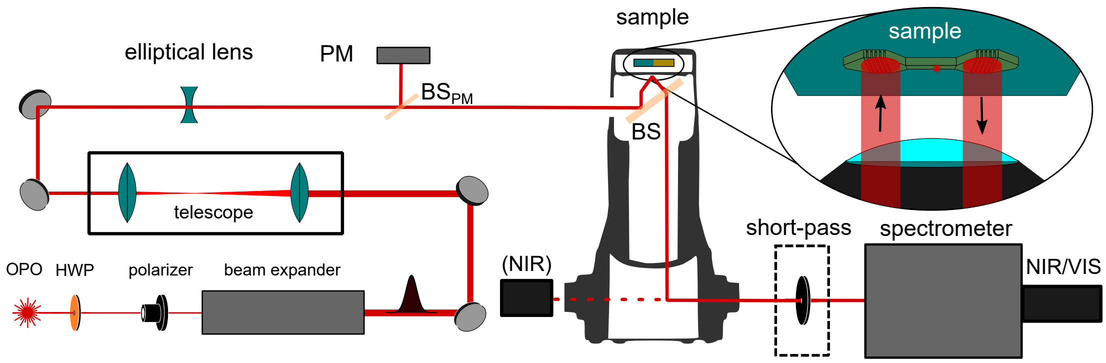

Supplementary Fig. 3. Experimental setup schematic. The source is an Optical Parametric Oscillator (OPO) (λ_p = 1320 nm, FWHM: 200 fs; repetition rate: 80 MHz). The output power is controlled via a motorized half-waveplate (HWP) placed before a polarizer. The beam is spatially shaped using a beam expander, telescope, and elliptical lens, so that its profile matches that of the input waveguide grating. A beamsplitter (BS_{PM}) and powermeter (PM) monitor the input power. A microscope holds the the WGs and HPICs. Light is delivered and collected to the sample via a 100× NIR microscope objective (Olympus, NA = 0.85) and BS. A short-pass filter (850 nm) is included in SHG experiments to filter out the NIR light. The scattered light is measured with an imaging spectrometer, using NIR (NIRvana) and VIS (PIXIS) cameras. An additonal NIR camera at a second output monitors alignment.

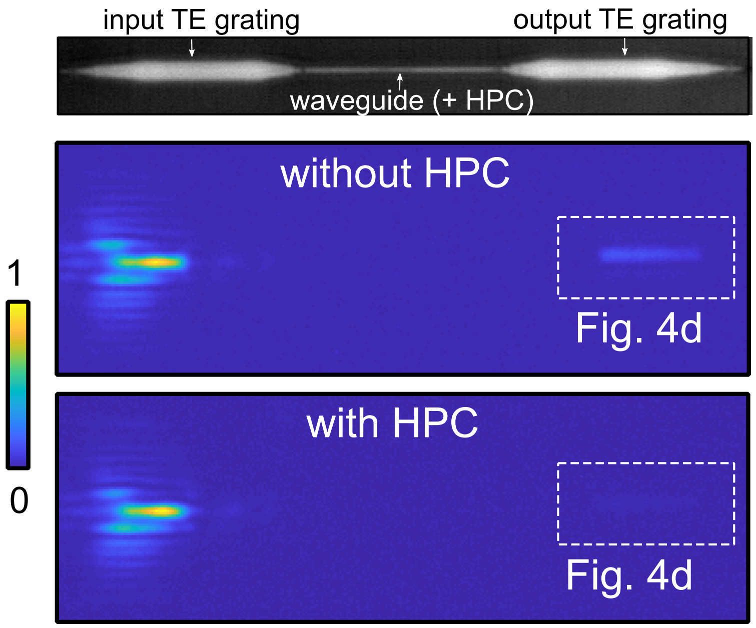

Supplementary Fig. 4. Zoom-out detail of Fig. 4(d) in the main manuscript, showing the comparable input scattering conditions used.

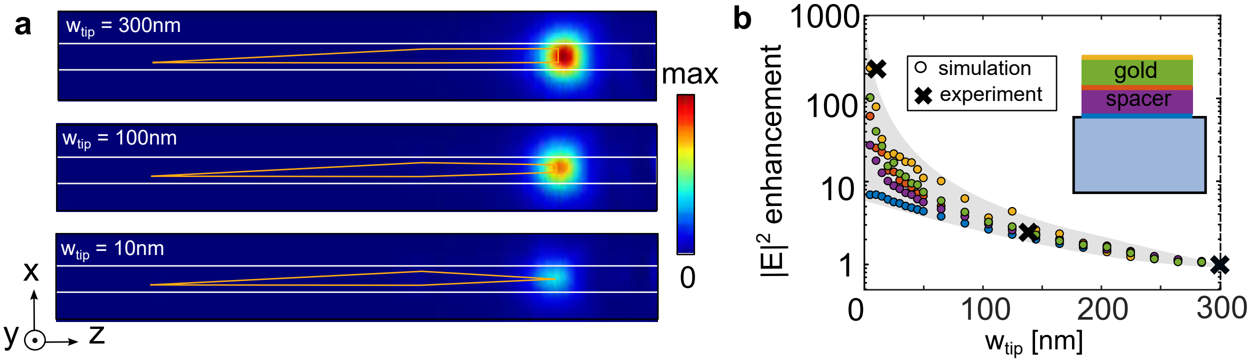

Supplementary Fig. 5. Calculations of the scattered pump light and intensity enhancement. (a) 3D FE calculations at $\lambda = 1.32\,\mu$m of the y-component of the Poynting vector, using a simulation space analogous to Fig. 3 of the main manuscript, for different tip sharpness as labelled ($w_{\rm tip}$). Slices are taken in the xz plane at a distance of $y = 660\,$nm from the top surface of the waveguide. Note the decreasing scattered intensity for increasing tip sharpness, for comparison with Fig. 5(a) and 5(b) of the main manuscript. Colorbar reprsents the y-component of the Poynting vector. (b) Calculations of enhancement at the tip, calculated with 3D FE calculations, showing the increasing local intensity for increasing tip sharpness for comparison with Fig. 6(b) of main manuscript. Calculations consider the average electric field at a tip of length $L_{\rm tip} = 3\,\mu$m and sharpness $w_{\rm tip}$ for the same input coupling conditions (corresponding the HTM mode in Fig. 2(b) (ii) of the main manuscript). We consider the average $|\mathbf{E}|^2$ on the top surface of the gold (yellow), inside the gold (green), on the bottom surface of the gold (orange), inside the spacer (purple), and on the bottom surface of the spacer (blue), each normalized to the case corresponding to the widest tip.

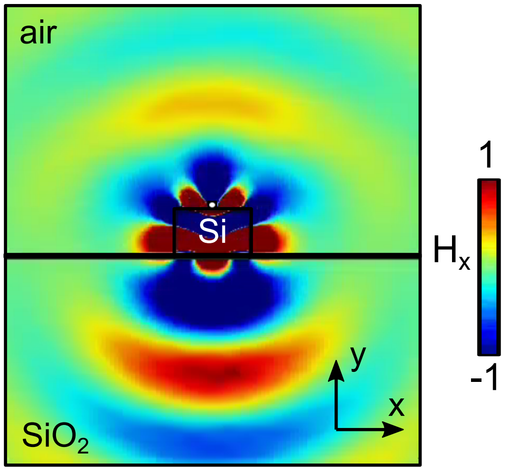

Supplementary Fig. 6. Calculations of the scattered light by the tip at the second harmonic wavelength. Cross section of 3D FE simulations of the transverse magnetic field radiated by a point source (wavelength: 660 nm; $n_{\mathrm{Si}} = 3.8365 + 0.015380i$ [1]; $n_{\mathrm{SiO2}} = 1.4563$ [2]) at a distance of 10 nm from the surface of an SOI waveguide, using the same simulations space as in Fig. 3 of the main manuscript, removing the plasmonic circuit. The fraction of power radiated upwards is 0.06%

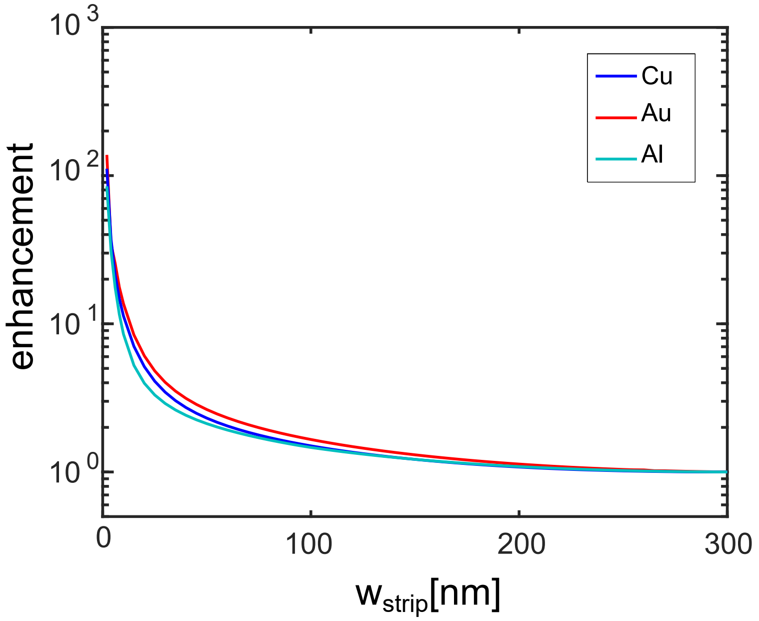

Supplementary Fig. 7. Calculations of the intensity enhancenment for different metals. Calculated enhancement (with respect to the widest strip width considered) as a function of strip width w_{strip} and for a focusser length of $3\,\mu$m (all other parameters are unchanged with respect to Fig. 6 in the main manuscript) for different metal films (red: gold [3]; blue: copper [3]; cyan: aluminium [4]), using the method outlined in [5]. The free space wavelength is $\lambda_0 = 1320\,\mathrm{nm}$.

Supplementary References

* alessandro.tuniz@sydney.edu.au

[1] D. E. Aspnes and A. A. Studna, "Dielectric functions and optical parameters of Si, Ge, GaP, GaAs, GaSb, InP, InAs, and InSb from 1.5 to 6.0 eV," Physical Review B **27**, 985–1009 (1983).

[2] I. H. Malitson, "Interspecimen comparison of the refractive index of fused silica," Journal of the Optical Society of America **55**, 1205–1209 (1965).

[3] P. D. Johnson and R. W. Christy, "Optical constants of the noble metals," Physical Review B **6**, 4370–4379 (1972).

[4] Aleksandar D Rakić, "Algorithm for the determination of intrinsic optical constants of metal films: application to aluminum," Applied Optics **34**, 4755–4767 (1995).

[5] L. Lafone, T. P. H. Sidiropoulos, and R. F. Oulton, "Silicon-based metal-loaded plasmonic waveguides for low-loss nanofocusing," Optics Letters **39**, 4356–4359 (2014).
